# ﻿*Larissimusnigricans* sp. nov. (Hymenoptera, Braconidae), a new reared species of a rare neotropical genus recovered through biodiversity inventory in Ecuador

**DOI:** 10.3897/zookeys.1156.101396

**Published:** 2023-03-24

**Authors:** Pomona Carrington-Hoekstra, Jose Fernandez-Triana, Lee A. Dyer, James Whitfield

**Affiliations:** 1 School of Integrative Biology, University of Illinois, Urbana, IL 61801 USA; 2 Canadian National Collection of Insects, Ottawa, ON K1A 0C6 Canada; 3 Department of Biology, University of Nevada, Reno, NV 89557 USA; 4 Department of Entomology, University of Illinois, Urbana, IL 61801 USA

**Keywords:** Arctiinae, bamboo, *Chusquea*, Erebidae, Lepidoptera, parasitoid

## Abstract

A new species of the rarely collected neotropical microgastrine braconid wasp genus *Larissimus* Nixon, represented previously by only a single described species, *L.cassander* Nixon, was recovered by the Caterpillars and Parasitoids of the Eastern Andes in Ecuador inventory project. *Larissimusnigricans***sp. nov.** was reared from an unidentified species of arctiine Erebidae feeding on the common bamboo species *Chusqueascandens* Kunth at the Yanayacu Biological Station near Cosanga, Napo Province, Ecuador. The new species is described and diagnosed from *L.cassander* using both morphological and DNA barcode data.

## ﻿Introduction

The braconid parasitoid wasp subfamily Microgastrinae currently contains 81 recognized genera and roughly 3,000 described species (Fernandez-Triana et al. 2020), although the true species richness may perhaps be over 45,000 species when undescribed species are included (Rodriguez et al. 2013). While clearly the ratio of undescribed to described species is large, this hyperdiverse group is nevertheless better understood than most in terms of higher classification and natural history (summarized by Whitfield et al. 2018). Undeniably, some of this understanding has resulted from focused historical specialization by taxonomists, as well as the ease of collecting adult species of the group using flight traps. Additionally, the rich data emerging from tropical inventories of caterpillars, their host plants, and parasitoids have provided an especially enlightening window into the ecology and diversity of the group (Forister et al. 2015).

The new reared species was discovered via the Caterpillars and Parasitoids of the Eastern Andes in Ecuador project, with fieldwork involving researchers from around the world for identification and description of host plants, caterpillars, and parasitoids. To date the project has produced 10,091 adult parasitoids, 3,648 of which are braconids belonging to 37 genera. For some genera, the project is greatly expanding knowledge of host biology over what was previously known from other regions.

The genus *Larissimus* was erected by Nixon (1965) for one neotropical species, *L.cassander* Nixon, from Brazil; the genus has been retained with essentially the same monotypic definition in subsequent treatments (Mason 1981; Whitfield et al. 2018; Fernandez-Triana et al. 2020). Penteado-Dias (1997) subsequently recorded the first host for *L.cassander*, *Bertholdia* sp. (Erebidae, Arctiinae) feeding on *Crotonfloribundus* (Euphorbiaceae). The newly reared species below is associated with a host from the same caterpillar subfamily; the presence of this genus in tropical forests beyond Brazil has been suspected (Whitfield et al. 2009) but not officially recorded with the described species.

## ﻿Materials and methods

### ﻿Field protocols for the Ecuador inventory

Adult parasitoids were reared from externally feeding larval Lepidoptera (i.e., caterpillars) that were collected by experienced parataxonomists, graduate students, undergraduate students, postdocs, Earthwatch volunteers, and principal investigators. To provide standardized estimates of caterpillar-parasitoid abundances and diversity, we employed survey methods currently in use at multiple sites across the Americas. Briefly, we document interaction diversity and collect all specimens within 5–30 m diameter plots along elevational gradients within a field site. This method includes intensive searches on specific host plants and documentation of caterpillar densities on individual plants, parasitoid loads on caterpillars, and quantitative data on species richness per leaf area. Plots were supplemented by general collecting of immature and adult Lepidoptera along the same elevational gradients. All collected caterpillars were reared using methods published elsewhere (Dyer et al. 2007; Wagner et al. 2021). Collecting and rearing took place continuously from 2000 to present, with a standard week consisting of four days of general collecting, 2–6 plots sampled, and daily rearing. Collections included caterpillars that were clearly parasitized.

Immature Lepidoptera were identified to family and to a morphospecies common name by field staff. Adult lepidopteran specimens (reared or collected as adults) were field-pinned, transported to the University of Nevada, then spread and curated using standard techniques. Vouchers examined by various taxonomic authorities, including newly established type specimens and undescribed species, are housed in collections of the taxonomists’ preference to facilitate further systematic studies of the material. Voucher specimens of the focal plants and novel host records were collected and pressed to ensure accurate taxonomic identification, then deposited at national herbaria. Each plant, caterpillar, and parasitoid specimen collected was registered as a unique record in a detailed database currently in use by a collaborative team across the Americas (Forister et al. 2015).

When wasps emerged from any stages of lepidopteran hosts, they were killed and preserved directly into 95% ethanol. Data labels were included in the specimen vials with the full locality, dates of collection and emergence, and the collector’s name. Rearing containers were checked frequently in order to find the parasitoids while they were still alive. Caterpillar remains were preserved when possible and were not detached from the substrate if they were attached.

### ﻿Specimen study

Examination and photography of the Ecuador specimens was conducted with a Leica M205 C stereomicroscope fitted with a five-megapixel Leica DFC 425 digital microscope camera. Image stacking was performed using a motor drive on the scope and Zerene Stacker software v. 2.0 (http://zerenestacker.com). The Brazil specimens were photographed with a Keyence VHX-1000 digital microscope, using a lens with a range of 13–130×. Image stacking was performed using the software associated with the Keyence system. Morphological terminology follows that in Huber and Sharkey (1993) and Fernandez-Triana et al. (2014). A single midleg was removed from each specimen and processed for COI DNA barcode identification using standard protocols (Ratnasingham and Hebert 2007, 2013).

### ﻿Descriptive taxonomy

The large body size, antennae with placodes mostly irregularly arranged on flagellomeres or in three disorganized rows, mostly highly polished body, large well-defined fore wing areolet, reduced and poorly set off hind wing vannal lobe, and elongate hourglass-shaped and relatively narrow first metasomal tergite combined with triangular second mediotergite clearly placed the species within *Larissimus* (Whitfield 1997), even without female genitalic characters available. The new species is conspicuously different from *L.cassander* and can be separated on the basis of color (mostly blackish dark brown with infuscate wings) and body size (over 5.0 mm, unusual for microgastrines) alone. While only male specimens were available, the available material of *Larissimus*–both described and undescribed–in collections shows little sexual dimorphism with respect to color. *Larissimuscassander* (Figs 3, 4) recorded from Brazil (states of Santa Catarina and São Paulo) is bigger (body length 7.0–8.0 mm, fore wing length 8.0–9.0 mm) and much lighter colored in both sexes, with most of its body orange-yellow or reddish yellow, including bright yellow pterostigma. The new species of *Larissimus* from Ecuador is smaller (body length 5.5 mm, fore wing length 5.4 mm) and much darker, with most of its body black to dark brown but with some areas white (most of T1 and all laterotergites plus bordering areas of tergites) and dark brown pterostigma. The two species can also be distinguished by 12% base pair differences in their corresponding DNA barcodes and also in biology (see below for details on hosts).

#### ﻿ Larissimus nigricans

Taxon classificationAnimaliaHymenopteraBraconidae

Carrington-Hoekstra & Whitfield sp. nov.

A8723596-4DB9-568E-BDE1-77CD13912ED9

https://zoobank.org/D333F68F-7921-4E3E-A9FC-6A4D23BD8E18

Fig. 1A–F

##### Description.

**Holotype male.** Body length 5.5 mm: fore wing length 5.4 mm.

**Figure 1.**
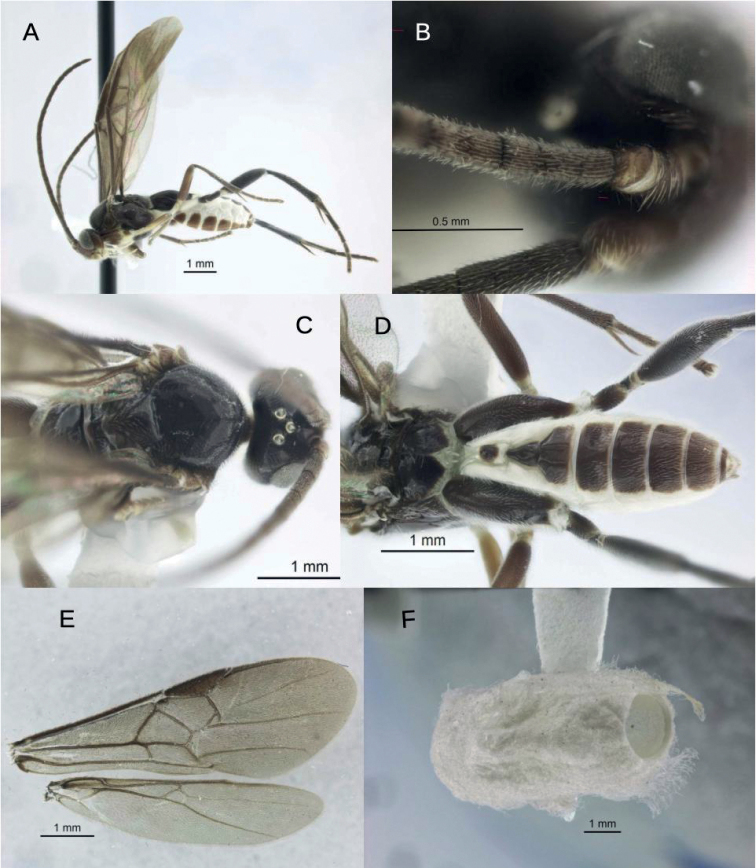
*Larissimusnigricans* sp. nov., male **A** lateral habitus **B** proximal regions of left antenna **C** dorsal view, head and mesosoma **D** dorsal view, propodeum and metasoma **E** wings **F** emerged cocoon.

***Color*** (Fig. 1A). Dark chestnut-brown, nearly black except: lighter brown clypeus, labrum, lateroventral portions of pronotum and propleuron, hind margin of propodeum, most of fore and mid legs and hind femur; whitish palpi, mid coxa, distoventral portions of hind coxa, anterior half and posterior margin of first metasomal tergite and lateral portions of metasomal tergites and sternites.

***Head*** (Fig. 1B, C). Antenna slender and roughly same length as body; placodes on flagellomeres roughly arranged in somewhat disorganized rows (typically three proximally). Face and eyes moderately setose, less so in an area posterior to the antenna and anterior to the ocelli. Labrum large and contrastingly colored (pale against a dark frons), with indentation dividing the labrum into a smaller dorsal section and larger ventral section. Ocelli arranged in low triangle (anterior edge of lateral ocelli on more or less same transverse line as posterior edge of anterior ocellus); lateral ocellus slightly truncated laterally due to overlapping cuticle.

***Mesosoma*** (Fig. 1A, C, D). Pronotum laterally mostly smooth and hairless with setae mostly confined dorsal portion; ventral groove broad and smooth, dorsal groove indistinct. Mesoscutum convex, nearly smooth over most of surface with denser setae and faint punctation anteriorly and laterally. Scutellum smooth, convex, and subtriangular. Mesopleuron hairless centrally, with smooth shallow concavity in posterior half. Propodeum smooth with sharply defined medial carina over entire length.

***Wings*** (Fig. 1E). Fore wing areolet of moderate size, strongly triangular. Vein 2r exiting the pterostigma at nearly a right angle (vs much more strongly angled to distal end in *L.cassander*) and straight to juncture with 1Rs. Height and length of triangular areolet nearly equal. Hind wing: Cu and cu-a bending slightly distally/”outward” at juncture with M + Cu. Vein 3M as strong as 2M. Vein r vaguely spectral or absent. 2r-m weak, unpigmented.

***Legs*** (Fig. 1A, D). Middle leg: inner tibial spur much longer than outer and nearly as long as basitarsus. Hind leg: proximal end of femur marked with a very narrow light band (matching trochanter); tibial spurs both long, inner longer than outer and roughly 0.75 as long as basitarsus.

***Metasoma*** (Fig. 1D, known only from male): tergite I smooth, more than twice as long as broad (narrowest just before midlength), with medial groove strongest anteriorly. Tergite II smooth, strongly triangular and longer than maximum width, more than twice as broad posteriorly as anteriorly. Laterotergites strongly whitish and matching lateral band of white on Tergite III and more posterior tergites. Tergite III rectangular over posterior half but with anterolateral corners angled; central third polished and slightly raised and set off from more setose lateral portions by longitudinal grooves. Tergites IV–V roughly rectangular with a small medial patch of hairlessness surrounded by light setae.

**Female.** Unknown.

##### Variation.

The two males available are extremely similar despite arising from different rearings in different years. The paratype male is slightly larger than the holotype.

##### Cocoon

**(Fig. 1F).** White, bluntly oval, spun from dense silk with loose strands externally.

##### Hosts.

**Figure 2.**
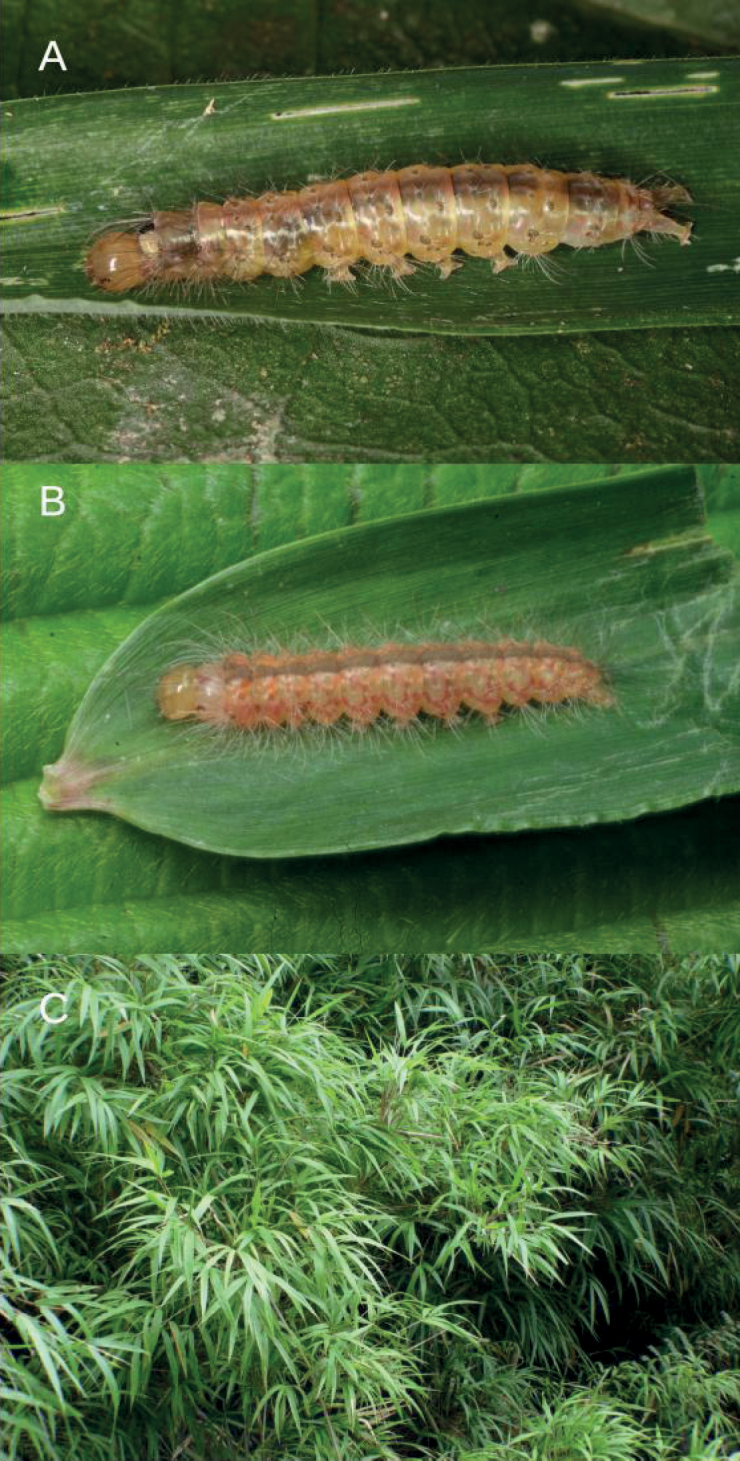
Host and host plant of *L.nigricans* sp. nov. **A** parasitized caterpillar of lithosiine (Arctiinae, Erebidae) moth on *Chusquea* leaf **B** unparasitized caterpillar of the same species, showing healthy color, and from which the adult was reared and identified as *Ardonea* sp. **C** a stand of *Chusqueascandens* Kunth from which the host caterpillars were reared.

Unidentified species of *Ardonea* Walker (Erebidae, subfamily Arctiinae, tribe Lithosiini) caterpillar (Fig. 2A, B) feeding on *Chusqueascandens* Kunth (Poaceae), a common and widespread Andean bamboo (Fig. 2C).

##### Material examined.

***Holotype*** male: Ecuador: Napo Prov., Yanayacu Biological Station, bamboo trail 2051, −0.5833, −77.8978, 218 m elev., collected 12 February 2011, rearing code 55036. ***Paratype***: same data as holotype but collected on yy road 2100, −0.5667, −77.8667, 21 April 2009, rearing code 38108. Both holotype and paratype deposited in Canadian National Collection of Insects, Ottawa (CNC).

##### Etymology.

From the Latin “nigricans”, meaning “blackish”. JFT and JBW have seen additional undescribed species of *Larissimus* (primarily in the Canadian National Insect Collection) with different color patterns, but not predominantly blackish ones.

##### Comments.

**Figure 3.**
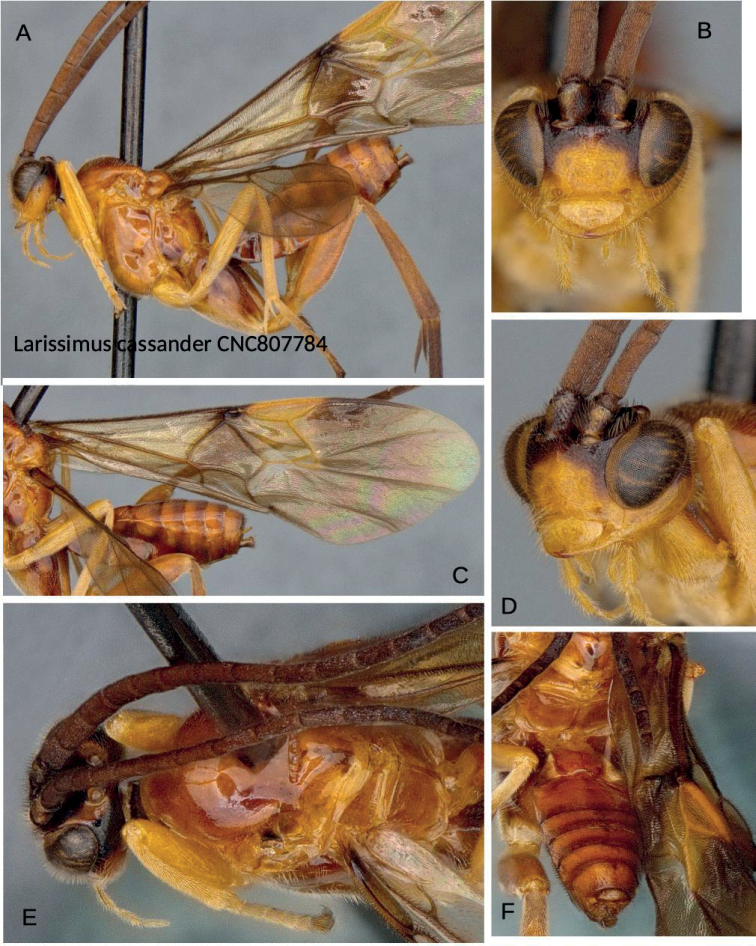
*Larissimuscassander* Nixon, male, Brazil **A** lateral habitus **B** head, frontal view **C** fore wing **D** head, view from left side, showing malar suture **E** dorsal view, head and mesosoma **F** dorsal view, metasoma.

Despite the dramatically different color combination and pattern, this new species is not strikingly different morphologically from *L.cassander*, at least based on the two males we have seen. Most structural differences are in minor shape proportions of structures (metasomal tergites narrower in the new species) and in wing vein angles (e.g., as mentioned above, compare angle of 2r in Figs 1E, 3C).

##### Molecular data.

Cytochrome c oxidase subunit 1 barcode sequence (sequence code BCNCC047-22 in the Barcode of Life (BOLD) database (Ratnasingham and Hebert 2007, 2013) consists of 614 bp and is 12% different in base pair identity from that of *L.cassander* (BOLD BIN BOLD:ABU6476).

**Figure 4.**
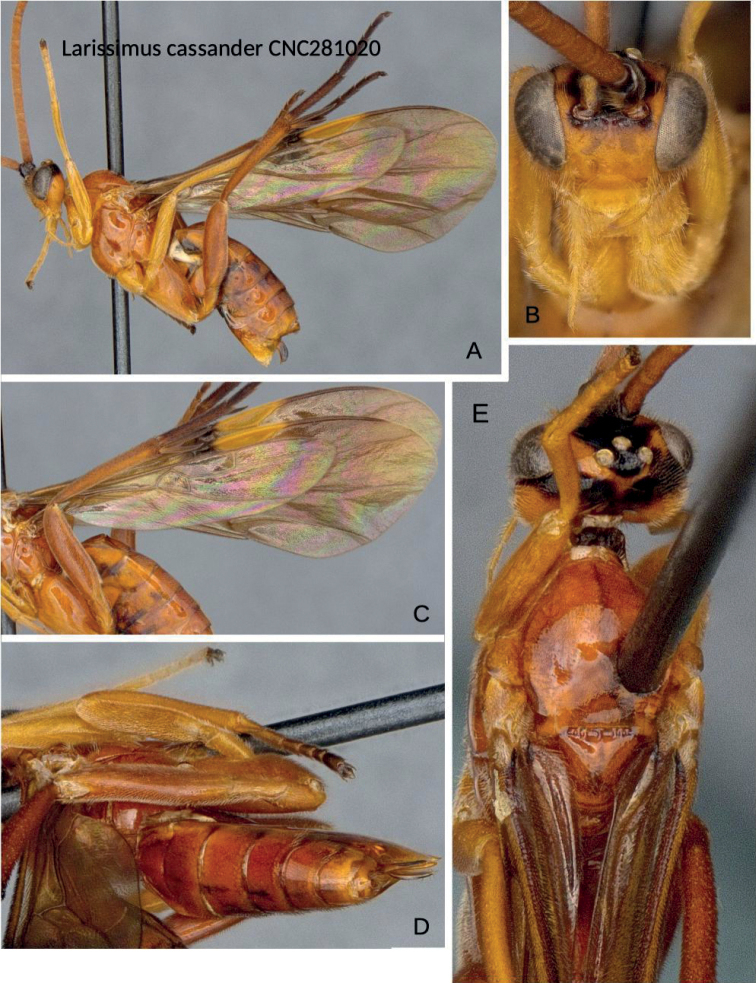
*Larissimuscassander* Nixon, female, Brazil **A** lateral habitus **B** head, frontal view **C** wings (especially showing hind wing) **D** dorsal view, metasoma **E** dorsal view, head and mesosoma.

## Supplementary Material

XML Treatment for Larissimus nigricans
